# Nurses’ willingness to work with COVID‐19 patients: The role of knowledge and attitude

**DOI:** 10.1002/nop2.674

**Published:** 2020-11-05

**Authors:** Abdulqadir J. Nashwan, Ahmad A. Abujaber, Ahmed S. Mohamed, Ralph C. Villar, Mahmood M. Al‐Jabry

**Affiliations:** ^1^ Department of Nursing Hazm Mebaireek General Hospital (HMGH) Hamad Medical Corporation (HMC) Doha Qatar; ^2^ Faculty of Nursing University of Calgary in Qatar Doha Qatar

**Keywords:** attitude, COVID‐19, nurses, SARS‐COV‐2, willingness

## Abstract

**Aim:**

This study aims to assess the role of nurses’ knowledge and attitude in relation to their willingness to work with patients diagnosed with COVID‐19 in Qatar.

**Design:**

A cross‐sectional study.

**Methods:**

A self‐administered, 35‐item online survey was circulated to the Registered Nurses working in Hamad Medical Corporation, the principal healthcare provider in Qatar.

**Results:**

A total of 580 attempts to complete the survey. Of them, 377 completed surveys with a response rate of 65%. Logistic regression was used to predict nurses’ willingness to work with patients with COVID‐19. Nurses’ knowledge level and monetary compensation that is associated with the work‐environment risk category were found to have a significant positive relationship with the nurses’ willingness to care for patients with COVID‐19 (*p* < .05). The findings of this study may help nursing leaders design educational programmes and remuneration models that may help boost nurses’ willingness to work with high‐risk patient groups, especially during a pandemic.

## 1 INTRODUCTION

In December 2019, a novel coronavirus that causes coronavirus disease 2019 (COVID‐19) was first detected in Wuhan, China (Imai, 2020; WHO, 2020). Similar to other previous respiratory coronavirus infections (e.g. SARS‐CoV and MERS‐CoV), patients presented with fever, dry cough, shortness of breathing and lung infiltration in the most severe cases (Gralinski & Menachery, 2020). There is still no definitive cure, and global pharmaceutical companies are rushing towards a vaccine.

Since the initial detection of the virus, more than 7.6 million cases of COVID‐19 have been confirmed worldwide. Qatar reported its first case on 27 February 2020. As of this writing (June 21, 2020), Qatar ranks fourth in the Eastern Mediterranean region having a total of 86,488 confirmed cases with 98 total deaths. In Qatar, nursing is the largest and the most diverse professional workforce in the healthcare system. Therefore, their role in defining the care and services is very instrumental. In Hamad Medical Corporation (HMC)‐ the principal healthcare provider in Qatar, more than 11,000 nurses and midwives working across different HMC hospitals, clinics, home care and residential services. In different studies, nurses are fearful of acquiring or being infected by a highly contagious virus. However, most are still willing to work and accept it as an obligation and part of their job despite the risks (Al‐Hunaishi et al., 2019; Liu et al., 2020; McMullan et al., 2016). The primary factor that can have an impact on the willingness of nurses is the hospital's preparedness plans that consider the safety of their staff members (McMullan et al., 2016). Al‐Hunaishi et al. suggested improving self‐efficacy through training can increase willingness to participate in a disaster, such as a flu‐pandemic (Al‐Hunaishi et al., 2019).

In 2004, research on the SARS epidemic has provided a preliminary understanding of some of the challenges related to looking after patients with SARS. Shiao et al. reported most Taiwanese nurses believed that they should be looking after SARS patients (87.8%) and only (25.9%) were intended to leave nursing due to the perceived personal risk (mostly due to lack of PPEs) (Shiao et al., 2007). Another study has investigated the relationship between hospital nurses’ attitudes towards SARS infection control measures and their professional care obligation. The findings showed that proper infection control measures were significant predictors of the nurses’ fulfilling of their professional care obligation (Tzeng, 2004).

On the other hand, the knowledge and attitude of nurses and other healthcare providers are expected to largely influence the degree of adherence to the proper use of personal protective measures and ultimately will be reflected on clinical outcomes for patients with COVID‐19.

Several studies were conducted on the awareness, knowledge, attitude and practices of nurses during the COVID‐19 pandemic. In Iran, Nemati et al. measured the awareness level among nurses. More than half of nurses (56.5%) had almost good knowledge about COVID‐19 related information (e.g. sources, transmission, signs and symptoms, prognosis, treatment and mortality rate). Mainly, most of the nurses were getting COVID‐19‐related information from the World Health Organization (WHO) official reports and the Ministry of Health (55%), social media (48%) and traditional media (42%). However, the overall knowledge score was not significantly different according to age, education level and work experience. The researchers suggested that providing more information might lead to better control of infectious diseases such as COVID‐19 (Nemati et al., 2020).

Another two studies were done in China (Liu et al., 2020) and India (Ip et al., 2015), where both of them targeted the public rather than nurses or healthcare providers. Overall, they found a moderate to adequate awareness related to transmission, symptoms and preventive measures for COVID‐19 infection. Therefore, it is crucial to explore the impact of knowledge and attitudes on the willingness of nurses and other healthcare providers during the COVID‐19 pandemic.

## 2 METHODS

### 2.1 Ethical approval

The study was approved by the Medical Research Center (MRC‐05‐065) and in full conformance with principles of the “Declaration of Helsinki” for Good Clinical Practice (GCP). The online survey link was sent to all participants via their official corporate email, where it was accompanied by a research information sheet explaining the expectations. The co‐investigators collected the data; then, it was coded and double‐checked by the PI (the PI was the link between data and code list); the data were stored in the protected computer by Hamad Medical Corporation (HMC) in accordance with the corporate's policies and guidelines.

### 2.2 Online survey

This cross‐sectional study was conducted in Hamad Medical Corporation (HMC) in June 2020. The 35‐item self‐administered survey was adapted from Shi et al., (2020). The original version was a 33‐item, Likert‐style questionnaire which included different levels of questions about the knowledge and attitudes towards COVID‐19 among medical staff in psychiatric hospitals in China. Permission to use the tool was granted and obtained by the principal investigator.

The survey link was circulated to all the nursing services at HMC; approximately 11,000 nurses via the corporate nursing e‐mail group. We assigned a two‐week period for completing the survey. During this period, we counted how many persons clicked the link. The response rate (65%) was calculated based on the number of completed surveys (377) divided by the number of staff who opened the link (580). The survey includes the participants’ demographics: gender, age, marital status, living status, hospital, unit, job title, level of education and COVID‐19 risk category.

The participants were asked to report the source of information and any relevant official training they had received in relation to COVID‐19. They were also asked to evaluate their level of knowledge and confidence in protecting themselves and others while working with COVID‐19 patients. The options were categorized into a 5‐point Likert scale (completely disagree, disagree, neither agree nor disagree, agree and completely agree). Lastly, the participants were requested to express their willingness to take care of patients with the COVID‐19 if/when they have the opportunity.

### 2.3 Statistical analysis

Data were analysed for descriptive and inferential statistics at a confidence level of 95% using SPSS v25. The scale reliability was evaluated for internal consistency and revealed satisfactory reliability (α above 0.6). Logistic regression was used to identify the predictors for nurses’ willingness to work with patients with COVID‐19.

### 2.4 Outcome variable

The outcome variable in this study is the nurse's willingness to care for patients with COVID‐19. The variable is binary where [yes = 0] reflects staff willingness to work with COVID‐19 patients, while [No = 1] means that the staff is unwilling to work with COVID‐19 patients. The statistical model was designed to predict unwillingness [No = 1].

## 3 RESULTS

### 3.1 Participants’ characteristics

A total of 377 questionnaires were collected and included in the final analysis out of 580; the response rate was 65%. The mean age of participants was 35.5 years (*SD* 6.3). Of them, 61.5% are females (Table 1). 88.1% of the participants expressed their willingness to work with COVID‐19 patients compared with 11.9% who were unwilling.

**Table 1:** Demographic data of respondents (*N* = 377)

Demographics	*N*	(%)
Gender		
Male	139	36.9
Female	232	61.5
Not declared	6	1.6
Age	**Mean**	***SD***
	**35.53**	**6.32**
Marital status		
Single	81	21.5
Married	292	77.5
Others	4	1.1
Living status		
Living with children	56	14.9
Living with spouse	69	18.3
Living alone	109	28.9
Living with children and spouse	126	33.4
Living with parents, children, and spouse	17	4.5
HMC facility		
AWH	44	11.7
HGH	79	21.0
HMGH	111	29.4
WWRC	40	10.6
Others	103	27.3
Job title		
Registered Nurse	244	64.7
Charge Nurse	33	8.8
Others	100	26.5
Education level		
Diploma	41	10.9
BSN	300	79.6
MSN	36	9.5
Risk category*		
High risk (I)	46	12.2
Moderate (II)	84	22.3
Low (III)	247	65.5
Willingness to treat and/or care for patients with COVID‐19		
Yes	332	88.1
No	45	11.9

_Abbreviations: AWH, Al Wakra Hospital; HGH, Hamad General Hospital; HMGH, Hazm Mebaireek General Hospital; WWRC, Women Wellness and Research Center._

* _High risk (I): all staff who are directly exposed to COVID‐19‐positive cases and are at the highest risk areas. These include specific hospitals/wards and some quarantine zones that have positive cases. Moderate (II): all staff who are currently working across all our Quarantine zones with suspected COVID‐19 cases. These exclude those who fall under Category‐1 in Quarantine zones. Low (III): all staff who are indirectly supporting the COVID‐19 pandemic (e.g. office work)._

### 3.2 The reliability analysis

As the reliability of the tool was not reported by Shi et al., (2020) study. In this study, the scale was evaluated for reliability and internal consistency and revealed satisfactory results with α greater than 0.06 (Sekaran & Bougie, 2016). Nurses’ knowledge 6‐item subscale scored α 0.74 and nurses’ attitude towards infection control precautions in COVID‐19 facilities 5‐item subscale scored α 0.61 (Table 2). No item deletion was required. The remaining 24 items were including the participants’ demographics.

**Table 2:** The reliability analysis of the survey

No.	Item	Cronbach's Alpha
K1	You understand the relevant knowledge of COVID‐19.	0.707
K2	You are confident that you understand the risks of COVID‐19 epidemic for the patients and medical staffs.	0.740
K3	You are confident that you understand how to protect yourself and your patients during COVID‐19 epidemic.	0.806
K4	Hand hygiene includes either washing hands with soap and water, or the use of an alcohol‐based hand rub.	0.695
K5	All recommended PPE is readily available in your hospital.	0.571
K6	You are aware about the proper PPE doffing technique.	0.717
Knowledge		**0.739**
A1	Rubbing hands with an alcohol‐based hand rub when they are visibly soiled.	0.698
A2	Use of correct PPE eliminates the need for hand hygiene.	0.671
A3	It is inconvenient to use recommended PPE when taking care for patients with COVID‐19.	0.740
A4	Use of recommended PPE interfere with patient treatment and/or nursing care.	0.485
A5	You know when your patients are on COVID‐19 precautions.	0.517
Attitude		**0.610**

### 3.3 Predictors of nurses’ willingness

A total of 332 participants (88.1%) showed their willingness to work with COVID‐19 patients. Logistic regression showed (Table 3) that the level of knowledge and the risk category that the staff is exposed to are the key predictors for nurses’ willingness to work with COVID‐19 patients. Nurses with a higher level of knowledge were more willing to work with COVID‐19 patients (OR 0.874, CI 0.766–0.996). Interestingly, the risk category is a strong predictor for nurses’ willingness. Nurses who reported ranked their level of risk exposure as low are less willing to take care of patients with COVID‐19.

**Table 3:** Predictors of willingness to care for patients with COVID‐19

Willingness to care of patients with COVID‐19	B	Std. Error	*df*	Sig.	Exp(B)	95% CI	
Knowledge	−0.135	0.067	1	0.044	0.874	0.766	0.996
Risk Category (Low)	2.119	0.520	1	0.000	8.322	3.001	23.076
Risk Category (Moderate)	1.167	0.471	1	0.013	3.213	1.275	8.095
Risk Category (High)	0		0				

The analysis shows that people who categorized themselves as low risk are less willing to care for patients with COVID‐19 (OR 8.322, CI 3.001–23.076). This was found to be associated with the financial compensation that the organization announced to pay for the nurses based on the work‐environment risk category. By conducting Chi‐square association analysis, we found no significant association between other demographics and nurses’ willingness to care for patients with COVID‐19.

## 4 DISCUSSION

The study reveals a very important aspect of nurses’ willingness to manage high‐risk patients. The nurses who reported low‐risk category expressed less willingness to care for patients with COVID‐19. It is very important to indicate that this variable was adopted from the risk categorization that was used by the organization for monetary compensation purposes. The organization announced that the nurses who provide direct care to patients with COVID‐19 would have higher risk allowance than those indirectly provide care for patients with COVID‐19, for example, nursing support and admin staff. Accordingly, this may implicitly indicate a relationship between the level of monetary gain and the risk that the nurse is willing to expose him/herself to. Nevertheless, it is very difficult to draw a comparison between nurses who directly care for patients with COVID‐19 and others who are working with suspected cases or indirectly providing administrative support.

Dezzani et al. examined potential predictors of nurses’ intentions to work during the 2009 influenza A (H1N1) pandemic where nurses were significantly more likely to work if certain incentives were offered (e.g. risk allowance, family protection, priority for vaccination/antiviral treatment) (Martin et al., 2013). Another study revealed that the most influential factors that motivated people to work were feeling that they were being protected by their country, local government and hospital (Imai, 2020). A possible explanation of this phenomenon is that most the nurses who work in Qatar are expatriates and particularly from Asia (El‐Jardali et al., 2013). Very importantly, nurses from different countries may have different value sets that influence their motivation. For example, Asian nurses are mostly motivated by financial rewards and it is the main reason that makes them leave their countries to pursue better commercial packages overseas (Henderson & Tulloch, 2008). The findings of our study showed nurses are more willing to work with COVID‐19 patients when they are more knowledgeable and well‐compensated for the level of work‐environment‐related risks.

Nevertheless, an experiment was discovered that incentive compensation should reward specific strategies (must be clear and known in advance) to improve communication and engage employees which ultimately leads to enhance leaders’ efforts to improve teamwork in large organizations (Lazear, 2018). Also, incentives can have adverse effects on individual motivation, especially when extrinsic motivation significantly displaces the intrinsic motivation of the subject (Ryan & Deci, 2000).

In this study, we found out that more than half of the respondents (53%) reported their overall level of knowledge related to COVID‐19 as “competent” and 34.3% rated themselves as “proficient” (Table 4). The result is consistent with other studies (Huynh et al., 2020; Nemati et al., 2020; Nepal et al., 2020; Saqlain et al., 2020; Zhou et al., 2020) where most healthcare workers reported having good knowledge about COVID‐19. This high percentage of knowledge among nurses and midwives regarding COVID‐19 is due to the immense volume of information that was made public by social media. The use of the Internet has drastically increased over the years. It has been reported that the average hours spent on the Internet by an individual is 6 hr 49 min a day, 2 hr and 16 min on social media and that is before the COVID‐19 pandemic ("Digital 2019: Global Internet Use Accelerates," 2020). According to The New York Times, Internet traffic in America has drastically increased during the pandemic with people seeking more information on COVID‐19 (Times, 2020).

**Table 4:** Knowledge of nurses during the COVID‐19 outbreak

	*N*	(%)
Overall level of knowledge		
Novice	2	0.5
Advanced beginner	28	7.5
Competent	200	53
Proficient	129	34.3
Expert	18	4.7
Sources of knowledge		
Television	241	64
Newspaper	170	45
Internet	350	93
Medical journals	249	66
Hospital training programme	12	3
Others	26	7

In China, one online medical service had exceeded 4.26 million consultations on COVID‐19 from 22 January–25 February 25, which is a 278% increase (Sun et al., 2020). One of the strategies to stop the spread of the virus is knowledge and information; for this reason, authorized governing body, such as the WHO (World Health Organization), CDC (Communicable Disease Center) and MoPH (Ministry of Public Health Qatar), has been consistently uploading COVID‐19 facts through their websites and different social media platforms. In Qatar, nurses and midwives receive daily updates from the System‐Wide Incident Command Committee Chair (SWICC) through SMS and emails and are granted free access to online medical journals. Table 2 shows that most of the respondents in this study obtain information from the Internet (93%), medical journals (66%) and television (64%). This is also true in the study on the assessment of COVID‐19 knowledge among nurses in Iran where most information was obtained from the Internet: 55% from the World Health Organization (WHO) and their Ministry of Health, 88% from social media and 42% from traditional media. In comparison to one study in Taiwan, healthcare workers generally receive information about COVID‐19 through formal lessons which resulted in greater self‐confidence (Wang et al., 2020).

Different hospitals in Qatar have initiated programmes to prepare nurses for COVID‐19; however, only 12 out of 377 (3%) of our respondents have completed hospital‐based training programmes. A probable reason for this is that those who are less exposed did not find the urgency to attend such programmes or that the training was confined to nurses and midwives who are directly working with COVID‐19 patients. Still, the respondents claimed to have good knowledge and high willingness (88.1%) to treat and care for patients with COVID‐19 among nurses and midwives. This is also, in contrast to the study of Al Hunashi, et al. where training healthcare workers improve the willingness to participate in the pandemic crisis (Al‐Hunaishi et al., 2019).

The perception of occupational exposure may lead to an unwillingness to go to work, especially when they witness their colleagues acquire the disease (Al‐Hunaishi et al., 2019; Ip et al., 2015; Liu et al., 2020; McMullan et al., 2016). In Thailand, most healthcare professionals were not willing to accept new patients or take care of patients during the COVID‐19 pandemic (Apisarnthanarak et al., 2020). Because of fear of acquiring the virus, nurses and midwives may hesitate to provide the usual care or they minimize their caring hours. There have been studies on previous flu epidemics where an increase in absenteeism is noticeable among nurses and other health care workers, which may or may not be related to sickness (Considine et al., 2011; Ip et al., 2015; Seale et al., 2008, 2009). A systematic review and meta‐analysis showed that confidence in safety, risk perception, prior training, general and role knowledge and confidence in skills were proven facilitators for willingness to work during an influenza pandemic (Aoyagi et al., 2015). Unlike most of the earlier‐mentioned studies, no significant associations were found between demographics and staff willingness to care for patients with COVID‐19.

## 5 LIMITATIONS

There are two significant limitations in this study that could be addressed in future research—first, the possibility of selection bias. Although we sent the invitation to over 11,000 nurses via the corporate e‐mail, the respondents were, directly and indirectly, working with COVID‐19 patients. Second, the cross‐sectional nature of the study was limited, and self‐reported questionnaires are dependent on the participants' honesty as well as the validity and reliability of the tool.

## 6 CONCLUSION

In conclusion, nurses’ level of knowledge and work‐environment risk category was found to play a significant role in predicting nurses’ willingness to work with COVID‐19 patients. The findings of this study could inform the policymakers in HMC and other healthcare institutions in Qatar to invest in training and improving staff's knowledge and to consider satisfactory remuneration systems to boost their willingness to work in a hazardous environment.

## COMPETING INTERESTS

The authors declare that they have no competing interests.

## ETHICAL APPROVAL AND CONSENT TO PARTICIPATE

The study was approved by the Medical Research Center (MRC) – Institutional Review Board (IRB) at Hamad Medical Corporation (MRC‐05‐065).

## AUTHORS' CONTRIBUTIONS

AJN*, AAA*, ASM, RCV, MMA: Research design, Data collection, Statistical analysis, Literature search, Manuscript preparation.

## Data Availability

All data generated during this study are included in this published article.

## References

[nop2674-bib-0001] Al‐Hunaishi, W. , Hoe, V. C. , & Chinna, K. (2019). Factors associated with healthcare workers willingness to participate in disasters: A cross‐sectional study in Sana’a, Yemen. British Medical Journal Open, 9(10), e030547 10.1136/bmjopen-2019-030547 PMC6803075 31628126

[nop2674-bib-0002] Aoyagi, Y. , Beck, C. R. , Dingwall, R. , & Nguyen‐Van‐Tam, J. S. (2015). Healthcare workers' willingness to work during an influenza pandemic: A systematic review and meta‐analysis. Influenza and Other Respiratory Viruses, 9(3), 120–130. 10.1111/irv.12310 25807865 PMC4415696

[nop2674-bib-0003] Apisarnthanarak A. , Apisarnthanarak P. , Siripraparat C. , Saengaram P. , Leeprechanon N. , Weber D. J. (2020). Impact of anxiety and fear for COVID‐19 toward infection control practices among Thai healthcare workers. Infection Control & Hospital Epidemiology, 41, (9), 1093–1094. 10.1017/ice.2020.280 32507115 PMC7298087

[nop2674-bib-0004] Considine, J. , Shaban, R. Z. , Patrick, J. , Holzhauser, K. , Aitken, P. , Clark, M. , & FitzGerald, G. (2011). Pandemic (H1N1) 2009 influenza in Australia: Absenteeism and redeployment of emergency medicine and nursing staff. Emergency Medicine Australasia, 23(5), 615–623. 10.1111/j.1742-6723.2011.01461.x 21995477

[nop2674-bib-0005] Digital 2019: Global Internet Use Accelerates . (2020). Retrieved from https://wearesocial.com/blog/2019/01/digital-2019-global-internet-use-accelerates

[nop2674-bib-0006] El‐Jardali, F. , Murray, S. F. , Dimassi, H. , Jamal, D. , AbuAlRub, R. , Al‐Surimi, K. , & Dumit, N. Y. (2013). Intention to stay of nurses in current posts in difficult‐to‐staff areas of Yemen, Jordan, Lebanon and Qatar: A cross‐sectional study. International Journal of Nursing Studies, 50, 1481–1494. 10.1016/j.ijnurstu.2013.02.013 23545140

[nop2674-bib-0007] Gralinski, L. E. , & Menachery, V. D. (2020). Return of the Coronavirus: 2019‐nCoV. Viruses, 12(2), 135 10.3390/v12020135 PMC7077245 31991541

[nop2674-bib-0008] Henderson, L. N. , & Tulloch, J. (2008). Incentives for retaining and motivating health workers in Pacific and Asian countries. Human Resources for Health, 6(18), 1–20. 10.1186/1478-4491-6-18 18793436 PMC2569066

[nop2674-bib-0009] Huynh, G. , Nguyen, T. N. H. , Vo, K. N. , & Pham, L. A. (2020). Knowledge and attitude toward COVID‐19 among healthcare workers at District 2 Hospital, Ho Chi Minh City. Asian Pacific Journal of Tropical Medicine, 13(6), 260..

[nop2674-bib-0010] Imai, H. (2020). Trust is a key factor in the willingness of health professionals to work during the COVID‐19 outbreak: Experience from the H1N1 pandemic in Japan 2009. Psychiatry and Clinical Neurosciences, 74(5), 329–330. 10.1111/pcn.12995 32105381 PMC7228386

[nop2674-bib-0011] Ip, D. K. , Lau, E. H. , Tam, Y. H. , So, H. C. , Cowling, B. J. , & Kwok, H. K. (2015). Increases in absenteeism among health care workers in Hong Kong during influenza epidemics, 2004–2009. BMC Infectious Diseases, 15(1), 586 10.1186/s12879-015-1316-y 26715075 PMC4696217

[nop2674-bib-0012] Lazear, E. P. (2018). Compensation and incentives in the workplace. Journal of Economic Perspectives, 32(3), 195–214. 10.1257/jep.32.3.195

[nop2674-bib-0013] Liu, Q. , Luo, D. , Haase, J. E. , Guo, Q. , Wang, X. Q. , Liu, S. , & Yang, B. X. (2020). The experiences of health‐care providers during the COVID‐19 crisis in China: A qualitative study. The Lancet Global Health, 8(6), e790–e798. 10.1016/S2214-109X(20)30204-7 32573443 PMC7190296

[nop2674-bib-0014] Martin, S. D. , Brown, L. M. , & Reid, W. M. (2013). Predictors of nurses’ intentions to work during the 2009 influenza A (H1N1) pandemic. AJN the American Journal of Nursing, 113(12), 24–31. 10.1097/01.NAJ.0000438865.22036.15 24247662

[nop2674-bib-0015] McMullan, C. , Brown, G. D. , & O'Sullivan, D. (2016). Preparing to respond: Irish nurses' perceptions of preparedness for an influenza pandemic. International Emergency Nursing, 26, 3–7. 10.1016/j.ienj.2015.10.004 26597971

[nop2674-bib-0016] Nemati M. , Ebrahimi B. , Nemati F. (2020). Assessment of Iranian Nurses’ Knowledge and Anxiety Toward COVID‐19 During the Current Outbreak in Iran. Archives of Clinical Infectious Diseases, 15, (COVID‐19), 1–5. 10.5812/archcid.102848.

[nop2674-bib-0017] Nepal R. , Sapkota K. , Paudel P. , Adhikari B. , Adhikari K. , Sapkota K. , Nepal R. , Adhikari B.N. , Paudyal N. (2020). Knowledge, attitude and practice regarding COVID‐19 among healthcare workers in Chitwan, Nepal. Journal of Chitwan Medical College, 10, (3), 98–102. 10.3126/jcmc.v10i3.32064.

[nop2674-bib-0018] Ryan, R. M. , & Deci, E. L. (2000). Intrinsic and extrinsic motivations: Classic definitions and new directions. Contemporary Educational Psychology, 25(1), 54–67. 10.1006/ceps.1999.1020 10620381

[nop2674-bib-0019] Saqlain M. , Munir M.M. , Rehman S.U. , Gulzar A. , Naz S. , Ahmed Z. , Tahir A.H. , Mashhood M. (2020). Knowledge, attitude, practice and perceived barriers among healthcare workers regarding COVID‐19: a cross‐sectional survey from Pakistan. Journal of Hospital Infection, 105, (3), 419–423. 10.1016/j.jhin.2020.05.007. PMC7211584 32437822

[nop2674-bib-0020] Seale, H. , Leask, J. , Po, K. , & MacIntyre, C. R. (2009). "Will they just pack up and leave?"–attitudes and intended behaviour of hospital health care workers during an influenza pandemic. BMC Health Services Research, 9(1), 30 10.1186/1472-6963-9-30 19216792 PMC2661074

[nop2674-bib-0021] Seale H. , MacIntyre C.R. , Booy R. , Leask J. (2008). Knowledge, Attitudes and Intended Behaviour of Hospital Health Care Workers Around Pandemic Influenza. International Journal of Infectious Diseases, 12, e435 10.1016/j.ijid.2008.05.1274.

[nop2674-bib-0022] Sekaran, U. , & Bougie, R. (2016). Research Methods for Business: A Skill Building Approach, Hoboken, NJ: John Wiley & Sons.

[nop2674-bib-0023] Shi Y. , Wang J. , Yang Y. , Wang Z. , Wang G. , Hashimoto K. , Zhang K. , Liu H. (2020). Knowledge and attitudes of medical staff in Chinese psychiatric hospitals regarding COVID‐19. Brain, Behavior, & Immunity ‐ Health, 4, 100064 10.1016/j.bbih.2020.100064. PMC7138160 32289123

[nop2674-bib-0024] Shiao J.S.C. , Koh D. , Lo L.H , Lim M.K. , Guo Y. L. (2007). Factors Predicting Nurses' Consideration of Leaving their Job During the Sars Outbreak. Nursing Ethics, 14, (1), 5–17. 10.1177/0969733007071350 17334166

[nop2674-bib-0025] Sun, S. , Yu, K. , Xie, Z. , & Pan, X. (2020). China empowers Internet hospital to fight against COVID‐19. The Journal of Infection.81(1), e67–e68. https://pubmed.ncbi.nlm.nih.gov/32251688/. 32251688 10.1016/j.jinf.2020.03.061 PMC7129532

[nop2674-bib-0026] Times, T. N. Y. (2020). The Virus Changed the Way We Internet. Retrieved from https://www.nytimes.com/interactive/2020/04/07/technology/coronavirus-internet-use.html

[nop2674-bib-0027] Tzeng H.M. (2004). Nurses’ Professional Care Obligation and Their Attitudes Towards SARS Infection Control Measures in Taiwan During and After the 2003 Epidemic. Nursing Ethics, 11, (3), 277–289. 10.1191/096733004ne695oa. 15176641

[nop2674-bib-0028] Wang P.W. , Lu W.H. , Ko N.Y. , Chen Y.L. , Li D.J. , Chang Y.P. , Yen C.F. (2020). COVID‐19‐Related Information Sources and the Relationship With Confidence in People Coping with COVID‐19: Facebook Survey Study in Taiwan. Journal of Medical Internet Research, 22, (6), e20021 10.2196/20021. 32490839 PMC7279044

[nop2674-bib-0029] WHO (2020). Novel Coronavirus‐China, Geneva, Switzerland: World Health Organization. cited January, 20.

[nop2674-bib-0030] Zhou, M. , Tang, F. , Wang, Y. , Nie, H. , Zhang, L. , You, G. , & Zhang, M. (2020). Knowledge, attitude and practice regarding COVID‐19 among health care workers in Henan, China. Journal of Hospital Infection,105(2), 183–187. https://www.journalofhospitalinfection.com/article/S0195-6701(20)30187-0/fulltext 10.1016/j.jhin.2020.04.012 PMC7194961 32278701

